# Tolvaptan-induced isolated elevation of bilirubin in a patient with Gilbert syndrome

**DOI:** 10.1177/2050313X231169841

**Published:** 2023-04-29

**Authors:** Matthew Nguyen, Beshoy T Yanny, Tai LD Truong, Hongyu Zhao, Ramy Hanna

**Affiliations:** 1 Division of Nephrology, Hypertension & Kidney Transplantation, Department of Medicine, University of California, Irvine Medical Center, Irvine, CA, USA; 2 Vatche & Tamar Manoukian Division of Digestive Diseases, Department of Medicine, University of California, Los Angeles, Los Angeles, CA, USA

**Keywords:** Tolvaptan, Gilbert’s syndrome, autosomal dominant polycystic kidney disease

## Abstract

Tolvaptan is the current standard of treatment for autosomal dominant polycystic kidney disease. It operates by acting on V2 receptors and blocks vasopressin interactions, causing a reduction in the rate of renal cyst growth and preserving kidney function. The current known risks of tolvaptan involve a serious liver injury characterized by an elevation in total bilirubin and alanine transaminase and aspartate transaminase levels. In this report, we document a unique liver injury characterized by an elevated bilirubin with normal alanine transaminase and aspartate transaminase levels in a patient who is homozygous for the UGT1A1 consistent with Gilbert syndrome.

## Introduction

Autosomal polycystic kidney disease (ADPKD) is the most common monogenic kidney disease and the fourth leading cause of end-stage kidney disease.^1,2^ Patients with ADPKD have disruption of the polycystin 1 (PKD1) and polycystin 2 (PKD2) genes, causing increased proliferation, chloride-driven fluid secretion, and release of proinflammatory cytokines by the vasopressin-sensitive tubular epithelial cells in the distal nephrons and collecting ducts, and therefore, increasing the development of cysts and destruction of the renal parenchyma.^3^

Vasopressin has been shown to promote renal cyst proliferation by upregulation of the cyclic adenosine-3′,5′-cyclic monophosphate pathway (cAMP).^4^ Regulation of the V2-receptor blockades has been shown to prolong survival in rodent models by reducing cyst burden and protecting kidney function^5^ tolvaptan, a vasopressin antagonist, has been shown to reduce the decline in estimated glomerular filtration rate (GFR) by reducing cyst growth in patients with early ADPKD (estimated clearance, estimated clearance, ⩾60 mL/min).^6^ While beneficial, the use of tolvaptan has also been linked to liver injury that is reversible upon cessation of the drug.^7^ In the tolvaptan in Later-Stage Autosomal Dominant Polycystic Kidney Disease (REPRISE) Trial, tolvaptan was found to be efficacious in slowing the rate of decline in GFR by a difference of 1.27 mL/min per 1.73 m^2^ compared with the placebo group. However, elevations in aminotransferase levels three times the upper limit were also found but returned to normal limits with cessation of the drug. However, no patients were reported to have concurrent elevations in bilirubin levels twice the normal limits.^8^

We present an interesting case of isolated increase in bilirubin in a female patient with Gilbert’s syndrome after being on tolvaptan for 4 months for autosomal polycystic kidney disease. The patient developed increased bilirubin throughout the course with no symptoms and normal liver enzymes (alanine transaminase (ALT), aspartate transaminase (AST), alkaline phosphatase). After stopping tolvaptan, liver function tests (LFTs) showed improvement in bilirubin (T Bili) levels.

## Case presentation

A 20-year-old Caucasian Hispanic female with no significant past medical history and a family history of a mother and grandmother with polycystic kidney disease presented to the clinic for review of abnormal kidney ultrasound (US) results concerning for polycystic disease. At the time of her initial visit, she was not taking any medications and had no history of smoking, alcohol use, or illicit drug use. Vital signs in the clinic were within normal limits except for a mildly elevated blood pressure of 134/90. Initial labs were within normal limits with baseline creatinine at 0.5 and magnetic resonance imaging (MRI) of the abdomen showing numerous simple and complex cysts in both kidneys, most suggestive of autosomal dominant polycystic kidney disease (ADPKD). Given her family history, and the rapid onset of disease progression, the patient was clinically diagnosed with ADPKD type 1. Genetic testing for ADPKD was recommended, however, not completed due to lack of coverage by her insurance plan. Besides lifestyle recommendations of dietary interventions and hydration, Jynarque (tolvaptan) was also recommended given the recent and encouraging results of the REPRISE trial. Risks of liver toxicity, adverse effects, and benefits were also provided to the patient. The patient was agreeable to begin tolvaptan at 60 mg daily with biweekly follow-ups for 2 weeks with the following labs: comprehensive metabolic panel (CMP), cystatin c, uric acid, phosphorus, and magnesium.

At baseline, her ALT was 20, AST was 21, total bilirubin was 0.4, hemoglobin 13.0, albumin 4.1, basic metabolic panel (BMP), and complete blood count (CBC) were within normal limits. At her 2-week follow-up, her ALT and AST were unchanged at 22 and 19, respectively, and her total bilirubin was 0.5. All other labs were within normal limits. She endorsed polyuria and polydipsia concurrent with tolvaptan therapy, but the patient stated she had been hydrating well and tolerating treatment regimen. At 4-week follow-up, her ALT and AST were still within normal limits (28 and 25, respectively), however, her total bilirubin had increased to 1.1 unit. On the 4th week visit, she stated having no symptoms and a liver US was ordered. By 2 months’ follow-up, ALT and AST were unchanged at 12 and 17, respectively, but total bilirubin had increased to 2.2. Liver US showed mild heterogeneity and increased echogenicity in the liver. There were no focal hepatic masses or fat sparing. The gallbladder was distended and had 0.2-cm-thick walls. There was no evidence of gallbladder sludge, shadowing of gallstone, or sonographic Murphy’s sign. The biliary tree had a 0.3-mm diameter common bile duct, but no intrahepatic biliary duct dilatation. Albumin stayed above 4.0 throughout the whole course.

At this time, the tolvaptan was stopped. After 3 days of stopping the medication, repeat liver function tests revealed a decrease in total bilirubin from 2.2 to 1.7 with repeat test showing 1.6 after 2 weeks. At this time, conjugated bilirubin was ordered with a total bilirubin showing 0.4 and 1.9, respectively. A repeat conjugated bilirubin a month later shows a conjugated bilirubin of 0.3 and a total bilirubin of 1.4. Further gastrointestinal evaluation and genetic testing were positive for homozygosity of the UGT1A1 gene. The combination of unconjugated bilirubin predominance on blood draw in association with genetic testing is consistent with Gilbert’s syndrome, at which time her total bilirubin decreased to 1.2. She denied any history of liver disease, family history of liver disease, or complications of liver disease. During this time all other labs were within normal limits.

## Discussion

Tolvaptan works on V2 receptors in the collecting tubules by antagonizing the effects of vasopressin. It prevents the growth of renal cysts and worsening renal failure by causing water diuresis without impacting electrolyte excretion.^9^ Tolvaptan has been approved by the Food and Drug Administration (FDA) in April 2018 for use in patients with ADPKD with recommendations that it should not be prescribed to patients with preexisting moderate to severe liver disease.^10,11^

Tolvaptan causing potential liver injury has been well established in patients with ADPKD, including elevations in bilirubin levels. The TEMPO trial demonstrated that out of 961 patients, 1.5% of patients discontinued the drug due to liver damage, with 4.5% of these patients having a significant increase in aminotransferase values and 0.9% of these patients having an increase in bilirubin values.^6^ In a later trial (REPRISE) to assess the concern for hepatic injury for tolvaptan, it was found that approximately 10.9% of patients with ADPKD treated with tolvaptan had adverse hepatic events compared with untreated ADPKD patients (5.3%). The most adverse effect is an increase in the AST levels 3 times that of the upper normal limit followed by resolution of levels upon cessation of the drug.^8,12^ Given these concerns, the current recommendation for any ADPKD patient is to repeat AST, ALT, and bilirubin measurements at 2 and 4 weeks, and then monthly for the next 18 months and every 3 months thereafter.^13^

Our case is interesting as the patient does not follow the usual clinical presentation of hepatocellular injury and instead exhibited a cholestatic liver injury pattern. This is atypical with the use of tolvaptan and has been rarely reported. In most cases, patients exhibit a hepatocellular or mixed pattern of liver injury. To our knowledge, this is the first known case of isolated bilirubin abnormality in the setting of tolvaptan use. The workup for cholestatic injury did not show evidence of biliary obstruction, biliary dilatation, biliary strictures, or hepatocellular pattern or liver injury as evident by normal transaminases throughout treatment with tolvaptan. Genetic testing done later in the course reveals diagnosis of Gilbert’s syndrome in the patient, which is known to cause elevated indirect bilirubin levels and possible jaundice from an environmental stressor, most commonly in early adolescence.^14^ The cause of this syndrome is due to reduced glucuronidation of bilirubin and recurrent episodes of jaundice.^15^ It is likely that tolvaptan use may have exacerbated transient hyperbilirubinemia in the setting of her preexisting Gilbert syndrome that is corrected upon cessation of the drug. Multiple medications that use the Cytochrome P450 (CYP) system and require glucuronidation have been implicated in exacerbating Gilbert’s syndrome and worsening hyperbilirubinemia. In rodents, tolvaptan has been shown to use the glucuronide and CYP system therefore exacerbation of Gilbert’s syndrome is possible in this case.^16^ Our patient has not had any previous history of jaundice, nor has she had any previous blood work with elevated bilirubin. Therefore, this exacerbation was unique for her; it started after initiation of tolvaptan and rapidly improved after cessation of the drug, showing a temporal relationship with tolvaptan use and elevated bilirubin.

Compared with the REPRISE trial which reported no known elevations in bilirubin, this is the first known case where tolvaptan can raise bilirubin levels in the setting of patients with Gilbert syndrome. The prevalence of Gilbert syndrome is higher than that of ADPKD at a rate of 4%–16% compared with 0.027%, respectively.^14,17^ Given the prevalence of Gilbert syndrome being higher than ADPKD, it is imperative to assess the clinical and family history of the patient prior to starting tolvaptan. Furthermore, in this patient’s case, this unique idiosyncratic response between her condition and tolvaptan suggest further exploration of her genome for other physiological benefits or drug interaction. However, given the lack of studies regarding hyperbilirubinemia and tolvaptan use, our case encourages further studies regarding ADPKD patients with bilirubin-related comorbidities and tolvaptan use.

## Conclusion

**Figure 1.**
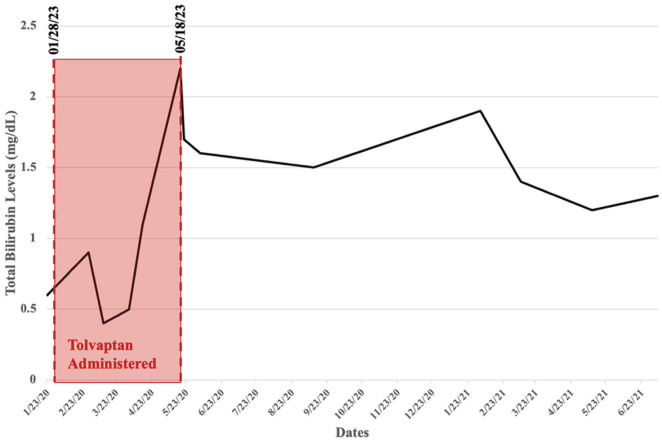
Total bilirubin levels measured over time with highlighted region during the time tolvaptan was administered.

Tolvaptan is the standard medication of choice used to treat patients with AKPDK. However, one major concern, although rare, is the development of hyperbilirubinemia for patients with Gilbert’s syndrome when associated with tolvaptan use. It is highly recommended to use tolvaptan for the appropriate group of patients based on FDA recommendations, while closely monitoring liver tests during treatment. Prescribers should obtain baseline liver function test prior to starting any patient on tolvaptan therapy and have subsequent follow-ups with repeat liver function test to assess for liver and biliary injury. This case suggests that tolvaptan may exacerbate Gilbert’s syndrome. More studies are needed to elucidate the safety of tolvaptan use in patients with inherited cholestatic liver disease, including Gilbert’s syndrome (Figure 1).
